# Supplementary material for: MetaRibo-Seq measures translation in microbiomes
Source: Nat Commun. 2020 Jun 29;11:3268. doi: 10.1038/s41467-020-17081-z (PMC7324362; doi:10.1038/s41467-020-17081-z)
Supplement: Supplementary file 10 — Supplementary Data 7 [file 41467_2020_17081_MOESM10_ESM.zip › File2/Confidence_High_Taxonomy/394877_out.krona.html]

Javascript must be enabled to view this page.

members
magnitude
magnitudeUnassigned
count
unassigned
taxon
rank

394877\_out

66

2
superkingdom
66

66
1239
phylum

186801
class
66

66
order
186802

family
186803
63

841
genus
1

360807
species

SRS016335\_contig\_number\_40580
1

62

SRS011084\_contig\_number\_14596SRS012849\_contig\_number\_33213SRS013098\_contig\_number\_44209SRS014235\_contig\_number\_26376SRS014412\_contig\_number\_12079SRS014736\_contig\_number\_7990SRS014923\_contig\_number\_28239SRS015217\_contig\_number\_29567SRS015431\_contig\_number\_16262SRS015578\_contig\_number\_31215SRS015579\_contig\_number\_10377SRS017433\_contig\_number\_17236SRS019161\_contig\_number\_16624SRS021484\_contig\_number\_17068SRS022137\_contig\_number\_19689SRS023346\_contig\_number\_6758SRS043411\_contig\_number\_15985SRS044535\_contig\_number\_21972SRS045004\_contig\_number\_32049SRS046717\_contig\_number\_10690SRS047014\_contig\_number\_654SRS047433\_contig\_number\_11540SRS049446\_contig\_number\_13401SRS050026\_contig\_number\_contig-100\_139.77601SRS050998\_contig\_number\_14153SRS053335\_contig\_number\_15530SRS054590\_contig\_number\_456SRS055982\_contig\_number\_12867SRS056273\_contig\_number\_24859SRS063190\_contig\_number\_11376SRS063370\_contig\_number\_12424SRS074586\_contig\_number\_3788SRS076804\_contig\_number\_18619SRS077294\_contig\_number\_17884SRS077502\_contig\_number\_13440SRS078176\_contig\_number\_15288SRS097920\_contig\_number\_25935SRS098073\_contig\_number\_19069SRS104084\_contig\_number\_13145SRS1041091\_contig\_number\_7884SRS1041095\_contig\_number\_5194SRS1041145\_contig\_number\_26136SRS104311\_contig\_number\_29860SRS104485\_contig\_number\_3654SRS1055049\_contig\_number\_11905SRS1055067\_contig\_number\_13904SRS1055076\_contig\_number\_9120SRS142712\_contig\_number\_5184SRS143466\_contig\_number\_32642SRS143780\_contig\_number\_13297SRS144506\_contig\_number\_54194SRS144714\_contig\_number\_29649SRS146764\_contig\_number\_25736SRS147425\_contig\_number\_11119SRS147614\_contig\_number\_1184SRS147766\_contig\_number\_7862SRS147919\_contig\_number\_17849SRS148196\_contig\_number\_40356SRS149325\_contig\_number\_16792SRS893252\_contig\_number\_19264SRS893295\_contig\_number\_13033SRS893341\_contig\_number\_14251
species
39491

186806
family
3

genus
1730
3

142586

SRS013951\_contig\_number\_25495SRS097958\_contig\_number\_23412
species
2

species

SRS148091\_contig\_number\_11147
1262883
1
